# Supplementary material for: Non-Invasive Intra-Abdominal Pressure Measurement by Means of Transient Radar Method: In Vitro Validation of a Novel Radar-Based Sensor
Source: Sensors (Basel). 2021 Sep 7;21(18):5999. doi: 10.3390/s21185999 (PMC8472078; doi:10.3390/s21185999)
Supplement: Supplementary file 1 [file sensors-21-05999-s001.zip › sensors-1354761-supplementary.pdf]

sensors

Supplementary Materials

Non-Invasive Intra-Abdominal Pressure Measurement by Means of Transient Radar Method: In Vitro Validation of a Novel Radar-Based Sensor

Salar Tayebi [1,*], Ali Pourkazemi [1], Manu L.N.G. Malbrain [2,3], and Johan Stiens [1]

1 Department of Electronics and Informatics, Vrije Universiteit Brussel, Brussels, Belgium; salar.tayebi@vub.be, apourkaz@etrovub.be, jstiens@etrovub.be
2 First Department of Anaesthesiology and Intensive Therapy, Medical University of Lublin, Lublin, Poland
3 Medical Department, Chief Medical Officer, AZ Jan Palfijn, Ghent, Belgium; manu.malbrain@janpalfijngent.be
* Correspondence: salar.tayebi@vub.be

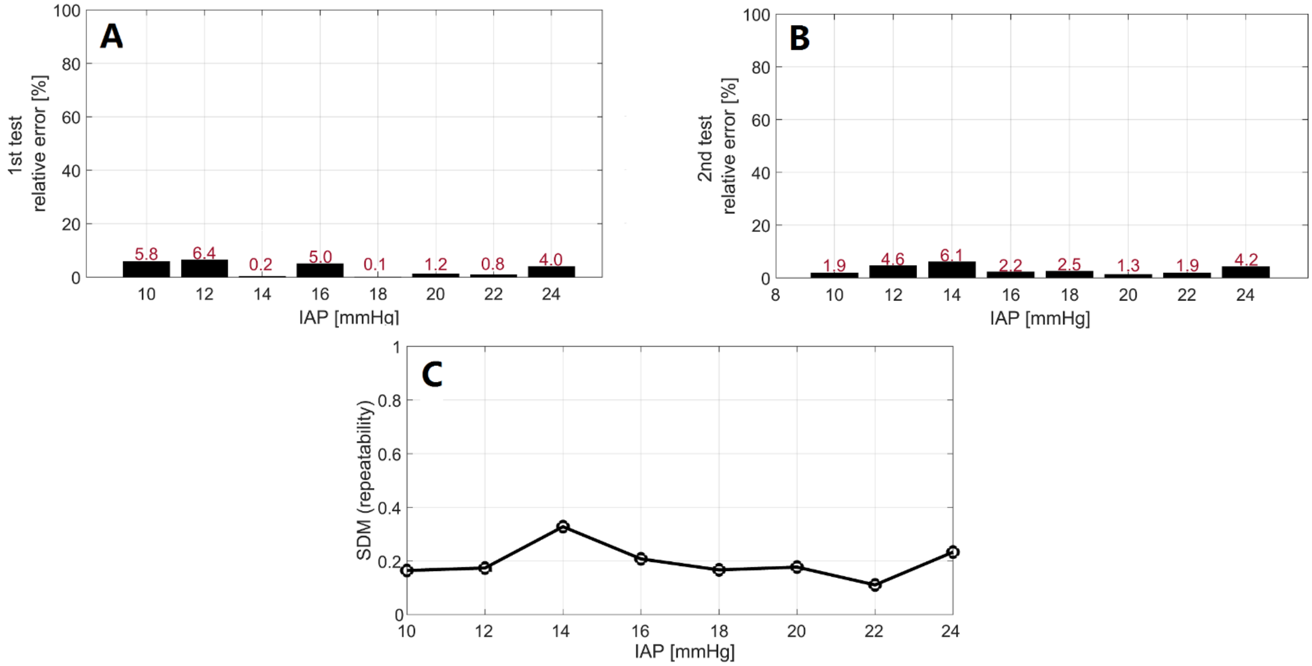

Figure S1. Relative error in addition to the standard deviation of mean (SDM).
